# A convenient broad-host counterselectable system endowing rapid genetic manipulations of *Kluyveromyces lactis* and other yeast species

**DOI:** 10.1186/s12934-024-02488-w

**Published:** 2024-07-26

**Authors:** Yanli Zheng, Yuhui Deng, Ping Hu, Shiqing Wang, Jiawen Wu, Siqi Luo, Lei Lei, Jiangke Yang, Wenfang Peng

**Affiliations:** 1 College of Life Science and Technology, Wuhan Polytechnic University, Wuhan, 430023 P. R. China; 2 State Key Laboratory of Biocatalysis and Enzyme Engineering, Hubei Engineering Research Center for Bio-enzyme Catalysis, Environmental Microbial Technology Center of Hubei Province, School of Life Sciences, Hubei University, Wuhan, 430062 P.R. China

## Abstract

Being generally regarded as safe, *Kluyveromyces lactis* has been widely taken for food, feed, and pharmaceutical applications, owing to its ability to achieve high levels of protein secretion and hence being suitable for industrial production of heterologous proteins. Production platform strains can be created through genetic engineering; while prototrophic cells without chromosomally accumulated antibiotics resistance genes have been generally preferred, arising the need for dominant counterselection. We report here the establishment of a convenient counterselection system based on a Frs2 variant, Frs2v, which is a mutant of the alpha-subunit of phenylalanyl-tRNA synthase capable of preferentially incorporating a toxic analog of phenylalanine, r-chloro-phenylalanine (4-CP), into proteins to bring about cell growth inhibition. We demonstrated that expression of Frs2v from an episomal plasmid in *K. lactis* could make the host cells sensitive to 2 mM 4-CP, and a Frs2v-expressing plasmid could be efficiently removed from the cells immediately after a single round of cell culturing in a 4-CP-contianing YPD medium. This Frs2v-based counterselection helped us attain scarless gene replacement in *K. lactis* without any prior engineering of the host cells. More importantly, counterselection with this system was proven to be functionally efficient also in *Saccharomyces cerevisiae* and *Komagataella phaffii*, suggestive of a broader application scope of the system in various yeast hosts. Collectively, this work has developed a strategy to enable rapid, convenient, and high-efficiency construction of prototrophic strains of *K. lactis* and possibly many other yeast species, and provided an important reference for establishing similar methods in other industrially important eukaryotic microbes.

**Supplementary Information:**

The online version contains supplementary material available at 10.1186/s12934-024-02488-w.

## Introduction

*Kluyveromyces lactis*, a non-conventional yeast, was for the first used as a source of lactase in milk products to overcome lactose intolerance of consumers in the early 1950s [1]. Since then, it has been studied and industrially applied for decades. As a host for heterologous protein production, *K. lactis* exhibits excellent fermentation characterics [1, 2]. For instance, it can be fermented at high cell densities using inexpensive standard yeast culture media. And the great capability of *K. lactis* in secretory protein production has allowed for reaching high extracellular protein concentrations in large-scale fermentations [1, 3]. These, together with its GRAS (generally regarded as safe) status, have promoted the safe use of *K. lactis* in various food, feed, and pharmaceutical applications [2].

The basic requirement for heterologous protein expression in *K. lactis* is the engineering of the cells to harbor an expression cassette of genes of interest. Most often, the expression cassette was introduced into cells in the form of either an episomal plasmid fragment or a chromosomal insert, where an antibiotic resistance marker was usually employed to provide selective pressure. The use of antibiotic resistance genes has been beneficial for initial strain construction and testing, but would not be practical in industrial protein production. Large-scale growth of cells in the presence of antibiotics can be extremely costly, and the addition of antibiotics is unfriendly for the safe use of *K. lactis* in applications concerning food, feed, or pharmacy. Moreover, the accumulation and subsequent limitation of viable selection markers can be a big problem if engineering of multi-gene pathways were in need. Therefore, it is always good to perform clean genetic manipulations with the help of counterselection for marker recovery.

Counterselection using the *Aspergillus nidulans amdS* gene has been employed for marker-free gene deletion in *K. lactis*. Interestingly, this marker can be used in both positive selection and counterselection schemes [4–6], thus suggestive of no need for a second selection marker. This can be beneficial for construction of plasmids with relatively smaller sizes. However, it was reported that during the counterselection process, three rounds of cell growth had to be performed to completely remove the chromosomally integrated *admS* gene, that is, at least 6–9 days were generally required for the isolation of a pure mutant [4]. Several recyclable marker genes, such as *URA3* [7–9], *LYS2* [10], and *MET15* [11], did have good performance in counterselection in yeast, but only in the corresponding auxotrophic yeast mutants, hence requiring prior engineering of the wild-type host cells. Although fortunately the development of the bacteriophage-derived Cre/*loxP* [12] and Flp/FRT [13] recombinase systems have allowed for recycling virtually any prototrophic markers, the repeated use of these systems would cause major chromosomal rearrangements [14]. In recent years, several PheS variants-based counterselection systems have been used in various bacterial species in a host-independent manner [15–22]. In *Escherichia coli*, where a PheS variant was first studied [23], PheS is the alpha-subunit of phenylalanyl-tRNA synthetase responsible for phenylalanine aminoacylation; whereas its derivative PheS* (PheS with T251A substitution) preferentially catalyzes the incorporation of an analog of phenylalanine, p-chloro-phenylalanine (4-CP), into proteins, resulting in cell death and therein providing robust counterselection pressure [24]. Conveniently, this method can be directly applied in prototrophic strains without any genetic modification.

We envisioned that genes coding for the alpha-subunit of phenylalanyl-tRNA synthetase could be functionally conserved in both prokaryotic and eukaryotic organisms, paving the possibility of establishing similar counterselection in *K. lactis*. To address the possibility, we attempted developing the *FRS2* gene of *K. lactis* GG799 [25], which encodes the alpha-subunit of phenylalanyl-tRNA synthetase Frs2, a eukaryotic PheS counterpart, as a counterselection marker by converting it to a conditional-lethal variant, Frs2v, mimicking the bacterial PheS* mutant. We experimentally evidenced the capacity of Frs2v for counterselection in *K. lactis*. With its assistance, rapid plasmid curing and scarless genetic manipulation of *K. lactis* have been efficiently accomplished, providing a versatile genetic manipulation toolkit for this yeast. Interestingly, we found that the counterselection was also efficiently functional for other yeasts including *S. cerevisia*e and *K. phaffii*. Considering that *FRS2* genes are present and highly conserved across yeast species, application of Frs2v-based counterselection in a broader host range of yeasts can be envisioned. This work would enable the development and further improvement of many yeasts as ideal production platforms for industrial applications, and has also provided an important reference for developing similar methods in other industrially important eukaryotic microbes.

## Materials and methods

### Strains, growth conditions and electroporation transformation

Yeast strains used or constructed in this work are listed in Table 1. Yeast cells were grown at 28ºC in a YPD medium (2% yeast extract, 1% bacto peptone, and 2% dextrose). If required, bleomycin was supplemented to the final concentration of 200 µg/mL for yeast and of 50 µg/mL for *E. coli*. Yeast competent cells were prepared and transformed with plasmids by electroporation using Bio-Rad Gene Pulser (0.1-cm gap cuvettes, 1.6 kV, 200 W, 25 µF) (Bio-Rad, Hercules, CA, USA) following the previously described methods [26]. Electroporated cells were incubated in a YPD medium with 500 mM sorbitol for 2 h at 28ºC prior to plating.

**Table 1:** Yeast strains used or constructed in this work

Strains	Genotype and features	Source
GG799	A wild-type *Kluyveromyces lactis* strain	[25]
INT	A derivative of GG799 with a DNA insert consisting of a *gfp* gene, a bleomycin resistance gene, and the Frs2v-expression cassette, at the *HAP1* locus	This work
∆*hap1*::*gfp*	A derivative of GG799 with a replacement of the *HAP1* gene by a *gfp* gene	This work
S288C	A wild-type *Saccharomyces cerevisia*e strain	[27]
GS115	A *Komagataella phaffii* wild-type strain	[28]

### Plasmid construction and DNA manipulation

The *E. coli*-*K. lactis* shuttle vector pEKb was generated by ligating three PCR fragments together, including an autonomously replicating sequence originated from *K. lactis* (panARS) being functional in diverse yeast species [29], the replication origin amplified from the *E. coli* pUC19 plasmid (pUC ori), and a bleomycin-resistant gene (BleoR) allowing for positive selection in both hosts. The panARS and the pUC ori were assembled together via splicing and overlap extension PCR (SOE-PCR) [30], and then ligated with the BleoR marker after BamHI and HindIII digestions. The *FRS2v* gene variant was created through the SOE-PCR method [30] and cloned into pEKb using the T5 exonuclease-dependent DNA assembly (TEDA) method [31], giving the pFrs2v plasmid. DNA fragment of the GFP expression cassette was amplified from a previous constructed plasmid [32] and used for generating the pFrs2v-GFP plasmid upon its insertion into pFrs2v.

DNA fragment used to facilitate homologous recombination and hence *HAP1* deletion was created by connecting the recombination arms, the *gfp* gene sequence, and the expression cassettes of the BleoR and the Frs2v markers. The fragments of each recombination arm were amplified from the genome of the wild-type GG799 strain, while that of the *gfp* gene sequence and the marker cassettes were amplified from the pFrs2v-GFP and pFrs2v plasmids, respectively. These fragments were connected by SOE-PCR [30].

All plasmids are listed in Table 2. All oligonucleotides were synthesized from GenScript (Nanjing, China) and listed in Table S3. Restriction enzymes and T5 exonuclease were purchased from New England Biolabs (Beijing) Ltd (Beijing, China).

**Table 2:** Plasmids used or constructed in this work

Plasmids	Genotype and features	Source
pEKb	An *Escherichia coli-Kluyveromyces lactis* shuttle vector containing the *E. coli* pUC19 replication origin and an autonomously replicating sequence of *K. lactis*; Bleo^r^	This work
pFrs2v	A pEKb derivative expressing a variant of Frs2	This work
pFrs2v-GFP	A pFrs2v derivative carrying a GFP-expression cassette	This work

### 4-CP sensitivity assay

Growth inhibition test was conducted for assaying the sensitivity of *K. lactis* strains to 4-CP. Overnight cultures of *K. lactis* strains were diluted in fresh YPD medium and growth to OD_600_ of 0.6. Then, each of the culture was serially 10-fold diluted up to 10^− 5^, and 5 µL of each dilution was spotted onto YPD4 agar plates where different concentrations of 4-CP were supplemented. The growth of each strain was photographically recorded after 72-hour incubation at 28ºC.

### Plasmid curing

Cells of the pFrs2v-GFP transformants were cultured in a liquid YPD medium containing bleomycin (YPDB). Afterwards, the growing cells were spread onto a YPD agar plate with 2.0 mM 4-CP (YPD4) while without bleomycin. Cells that formed colonies were regarded as those lost the plasmid.

### FACS analysis and florescence microscopy

Using similar protocols described previously [22, 32–34], cells were washed with phosphate buffered saline (PBS) twice, resuspended into PBS to a concentration of 10^7^/cells/mL, and analyzed with a Beckman CytoFLEX FCM (Beckman Coulter, Inc., USA) with PBS as the sheath fluid. The GFP fluorescent intensity of cells was detected with the FITC channel, 525/40 nm band pass. Fluorescent images of cells were obtained using a Zeiss laser-scanning microscope (Zeiss, German) with a 488-nm argon-ion laser.

### Construction and screening of *HAP1* deletant

The DNA fragment for *HAP1* replacement was introduced into yeast cells by electroporation. Electroporated cells were spread on an YPDB agar plate and incubated at 28ºC until colonies were seen. Cells of the transformants were grown up in a liquid YPDB medium, and then spread on a YPD4 agar. Cells that formed colonies were regarded as *HAP1* mutant candidates and subjected to colony PCR screening using primers listed in Table S2. The PCR products were analysed by agarose gel electrophoresis.

## Results and discussion

### Identifying the *K. lactis* GG799 Frs2 and constructing its Frs2v derivate

**Fig. 1:**
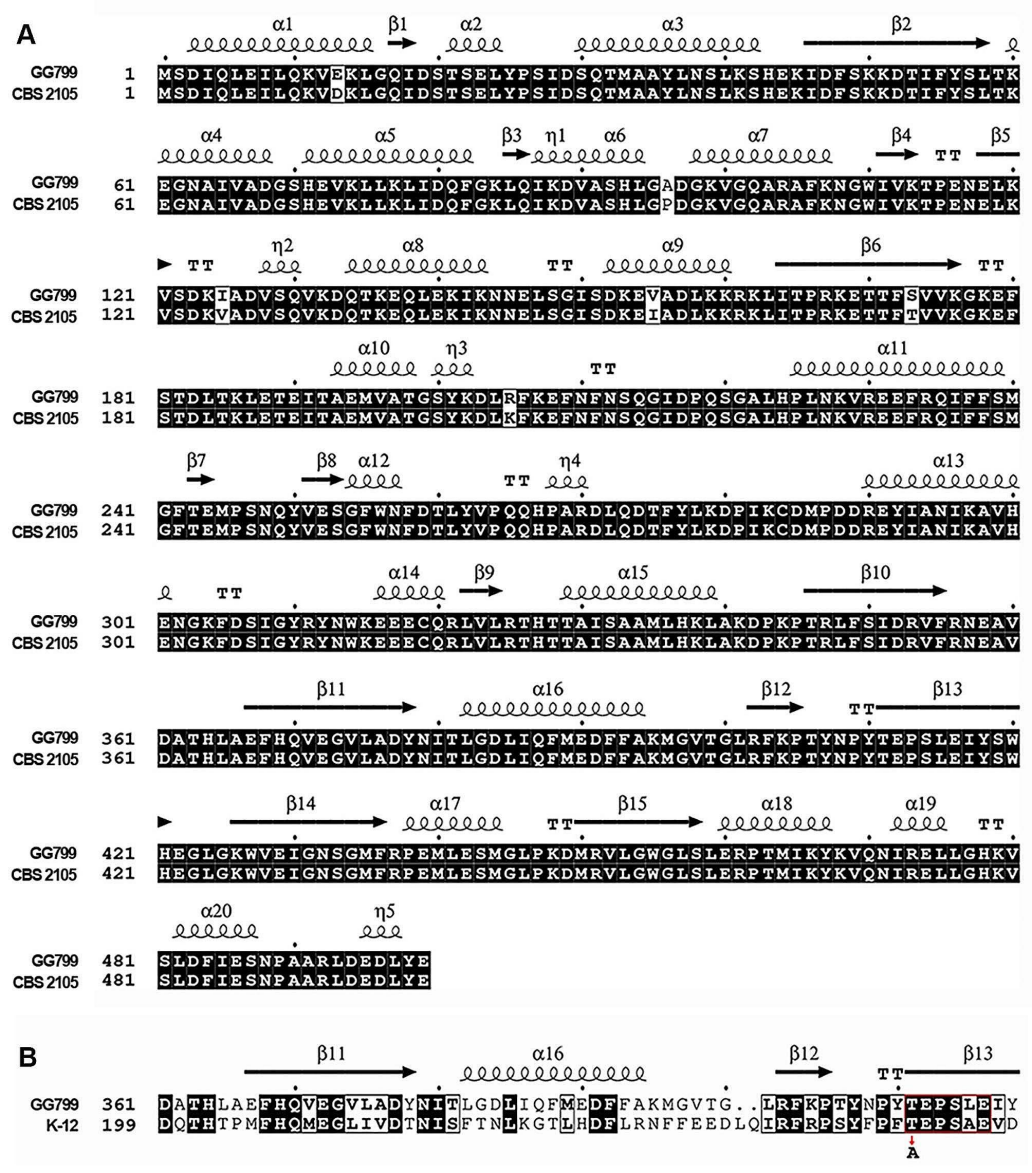
*K. lactis* Frs2 identification and Frs2v variant construction. (**A**) Amino acid sequence alignment of Frs2 proteins from two *K. lactis* strains, GG799 and CSB2105. (**B**) Amino acid sequence alignment of Frs2 from *K. lactis* GG799 and PheS from *E. coli* K-12. The conserved TEPSxE motif was highlighted with a red box, within which the T residual being subject to mutagenesis is indicated with a red arrow

The complete genome sequence of *K. lactis* GG799 has been publicly available [25], yet without annotation. In order to find out the sequence encoding the hypothetical PheS ortholog from the *K. lactis* GG799 genome, we performed local BLAST search with the NCBI BLAST+ (tblastn) program, taking amino acid sequence of the Phe-tRNA synthetase alpha-subunit (Frs2) from *K. lactis* strain CBS 2105 (GenBank: QEU58128) as an input. The BLAST result suggested a 1500-bp sequence spanning positions 2,348,421 to 2,349,920 of the chromosome F of *K. lactis* GG799 (Table 1), forming a complete ORF predictably coding for a 499-aa (amino acid) protein. Strikingly, the sequence of this peptide showed a near-100% identity to that of the Frs2 of *K. lactis* CBS2105 (Fig. 1A).

We then performed amino acid sequence alignment of the identified Frs2 with several bacterial PheS proteins, looking for the residue counterpart in Frs2 corresponding to the T251 residue of the *E. coli* PheS [24]. A conserved TEPSxE motif was seen in all the analyzed proteins, in which the T residue corresponds to the T251 of the *E. coli* PheS and the T411 of the *K. lactis* Frs2, respectively (Fig. 1B). Alike the T251A substitution previously made to the *E. coli* PheS [24], a T411A substitution was made to the *K. lactis* Frs2, giving Frs2v.

### Determining the sensitivity of Frs2v-expressing *K. lactis* cells to 4-CP

Previously, we and others have reported that higher levels expression of the mutated marker genes conferred much better performances in functional competition with the respective originals in respect of 4-CP incorporation and hence cell growth inhibition [20, 22, 35]. Therefore, a Frs2v-expression cassette with the strong constitutive P_*ADH1*_ promoter [36] was constructed and cloned onto pEKb, an *E. coli-K. lactis* shuttle vector expressing bleomycin resistance (BleoR), yielding the pFrs2v plasmid. Electroporation of this construct into *K. lactis* cells obtained as many transformants as that of the pEKb shuttle vector, showing a similar level of transformation efficiencies. From each transformation, three transformants were randomly chosen and individually cultured in liquid YPD media without 4-CP supplementation. No obvious growth retardation was observed for all of them (data not shown), suggestive of no influence of Frs2v expression on cell growth.

**Fig. 2:**
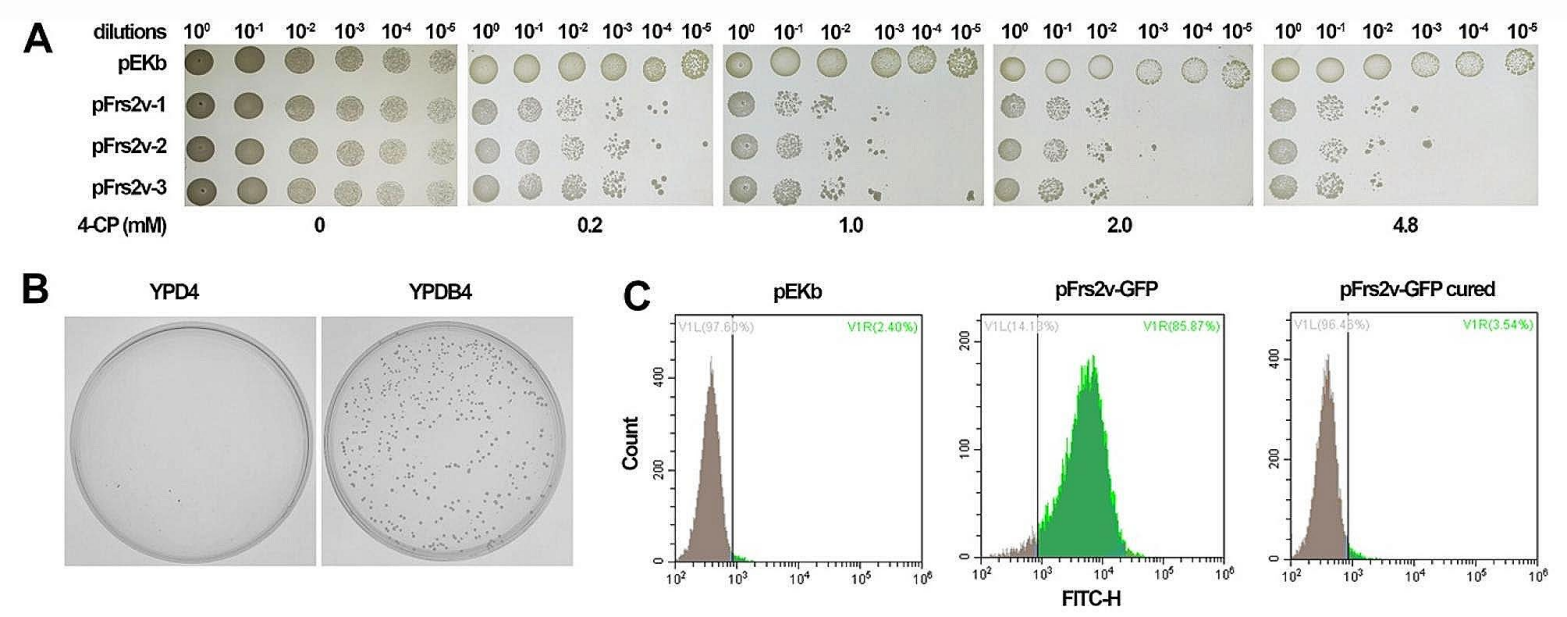
Plasmid curing using the Frs2v as a counterselection. **(A)** Growth inhibition of Frs2v-expressing strains by 4-CP. Cell cultures of transformants either carrying the shuttle vector pEKb, or the Frs2v-expressing plasmid pFrs2v, were serially 10-fold diluted. Dilutions were spotted onto YPD plates containing 4-CP at the indicated concentrations. (**B**) Examination of the counterselection effect of 4-CP on plasmid curing from the Frs2v transformants. (**C**) Detection of GFP signal in the cells of the pEKb transformant, and that of the pFrs2v-GFP transformant before and after plasmid curing via 4-CP counterselection

In order to examined the 4-CP sensitivity conferred by Frs2v to the *K. lactis* cells, we assayed growth inhibition of the pFrs2v transformants using YPDB plates individually containing 0, 0.2, 1.0, 2.0, and 4.8 mM of 4-CP. As shown in Fig. 2A, without 4-CP (0 mM), transformants of both pFrs2v and pEKb (as a reference) were normally and evenly grown up, including the 10^− 5^ dilutions. As the concentration of 4-CP was gradually increasingly supplemented, the inhibition effect on the growth of the pFrs2v transformants became clearer. Very few cells from the 10^3^-fold diluted pFrs2v transformants could grow up when spotted on YPD agar plates containing 2.0 mM of 4-CP (YPD4), whereas under the same condition the cells carrying pEKb kept growing up normally.

The results supported that Frs2v can be employed as an efficient counterselection marker for *K. lactis*, to the best of our knowledge, being the first eukaryotic counterpart of PheS for counterselection in yeast. Considering its no requirement for pretreatment of the wild-type host cells at all, it would represent one of currently the most convenient counterselectable systems for yeast.

### Attaining efficient plasmid-curing upon Frs2v-mediated counterselection

Next, we performed a plasmid-curing assay to verify the feasibility of Frs2v for counterselection. For a more straightforward observation of the removal of a Frs2v-bearing plasmid, a green fluorescent protein (GFP) expression cassette was inserted in the pFrs2v plasmid, generating pFrs2v-GFP. This would allow us to monitor the plasmid presence/absence by measuring the GFP fluorescence intensity through flow cytometry analysis. After electroporating the pFrs2v-GFP plasmid into *K. lactis* cells, many transformants appeared on a bleomycin-containing YPD plate (YPDB) without 4-CP supplementation. Cells of a randomly chosen transformant were transferred into a liquid 4-CP-containing medium of YPD (YPD4) or, of YPDB (YPDB4), for 48-hour incubation. They could not grow up in YPDB4 while grew normally in YPD4, and the growing cells could form colonies on agar plate of YPD4 but not YPDB4 (Fig. 2B).

In addition, signal of green fluorescence was detected in the cells before counterselection, whereas was not detectable in those suffered 4-CP treatment (Fig. 2C). These combined results suggested that the cells have completely discarded the Frs2v-expression plasmid and hence lost the resistance to bleomycin after a single round of 4-CP counterselection, demonstrating the effectiveness of the Frs2v marker for counterselection in *K. lactis*.

### Accomplishing scarless genetic manipulation of *K. Lactis* via Frs2v-based counterselection

**Fig. 3:**
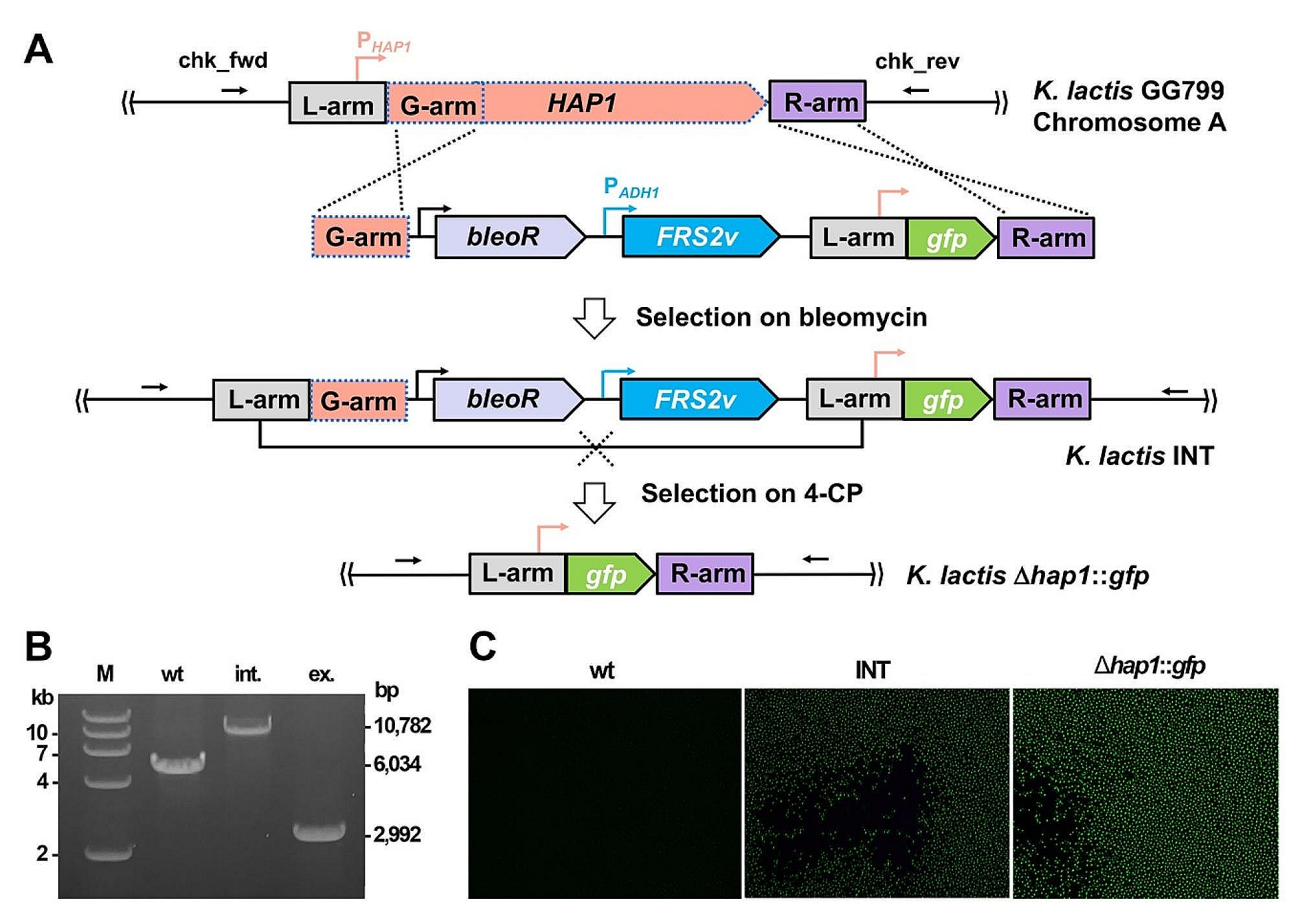
Scarless chromosomal modification with the assistance of Frs2v for counterselection. **(A)** Schematic showing design of the replacement of *HAP1* with *gfp*. A DNA stretch was designed to contain the bleomycin resistance gene (*bleoR*), a Frs2v-expression cassette, a *gfp* gene, and three arms (G-arm, L-arm, and R-arm) for homologous recombination. Transformants each with a DNA insert were selected on bleomycin; while excision of both the *bleoR* and the *FRS2v* markers was forced to occur in the form of recombination between two L-arms by 4-CP selection, giving the expected editing outcome of *HAP1* replacement by *gfp*. (**B**) PCR amplification verifying the strains of INT and Δ*hap1*::*gfp* using the primer set of chk_fwd and chk_rev indicated in (**A**). The predicted sizes of PCR products in the INT strain (int.) and the Δ*hap1*::*gfp* mutant (ex.), as well as the wild-type cells (wt), are shown. M, DNA size marker. (**C**) Microscopic evaluation of GFP fluorescence

Having constructed the high-efficiency Frs2v counterselection marker, we inferred its usefulness for scarless genetic manipulation of *K. lactis* and exemplified such by replacing the non-essential *HAP1* gene [37] with a *gfp* gene in an insertion-and-excision manner. As illustrated in Fig. 3A, the method simply included a single DNA fragment consisting of 3 recombination arms, the *gfp* gene, and a block of selection markers, that is, a gene arm (G-arm) composed of the first 948 bp coding sequences of *HAP1*, a left arm (L-arm) and a right arm (R-arm) corresponding to sequences immediately upstream and downstream of *HAP1*, respectively, sandwiching the *gfp* gene, and a block formed by two expression cassettes of selection markers, BleoR and Frs2v for positive selection and counterselection, respectively.

Upon introducing the DNA fragment into *K. lactis* cells, bleomycin-resistant transformants, designated as INT strains, were selected by the BleoR marker after integration of the entire DNA donor into the *HAP1* locus through recombination between the G-arm and the R-arm. The transformants could be seen on an YPDB agar plate after 2–3 days. Subsequently, the markers were excised via recombination between the homologous L-arms forced by 2.0 mM 4-CP selection via simply culturing the transformants in a liquid YPD4 medium for 2 days, leaving only the *gfp* gene at the *HAP1* locus. This gave the Δ*hap1*::*gfp* strain in which the transcription of the *gfp* gene was set to be driven by the native *HAP1* promoter. Both the integration (int.) and excision (ex.) were verified by PCR analyses using the primer set of chk_fwd + chk_rev, amplifying products with predicted sizes of 10,782 bp and 2,992 bp, respectively; whereas a 6,034-bp product was expected when the genome DNA of the wild-type cells was taken as a template (Fig. 3B). The fluorescence of the chromosomally incorporated GFP was observed using microscopy in both the strains of INT and Δ*hap1*::*gfp* (Fig. 3C). Much stronger fluorescence intensity was seen in the Δ*hap1*::*gfp* cells, presumably due to better cell growth after release of the bleomycin conferred selection pressure. These results demonstrated that an intended mutation could be precisely made to the *K. lactis* chromosome in a total of only 4–5 days. Conclusively, with the assistance of Frs2v-based counterselection, simplified, convenient, yet highly efficient genetic modifications of *K. lactis* genome can be readily attainable as designed.

We believe that, equipped with this counterselection system, genetic manipulation toolkits, such as the advanced CRISPR-Cas-based technologies, would generally perform better in yeast genome editing, being beneficial for the development and further improvement of yeast strains. For instance, its capacity in curing of the genome editing plasmid, a process reported to be tedious and time-consuming, can make the host cells be rapidly ready for the next round of editing immediately after the Frs2v-mediated counterselection, thus expediting multi-round genome engineering.

### Using the same *K. lactis* Frs2v for counterselection in yeasts other than *K. lactis*

**Fig. 4:**
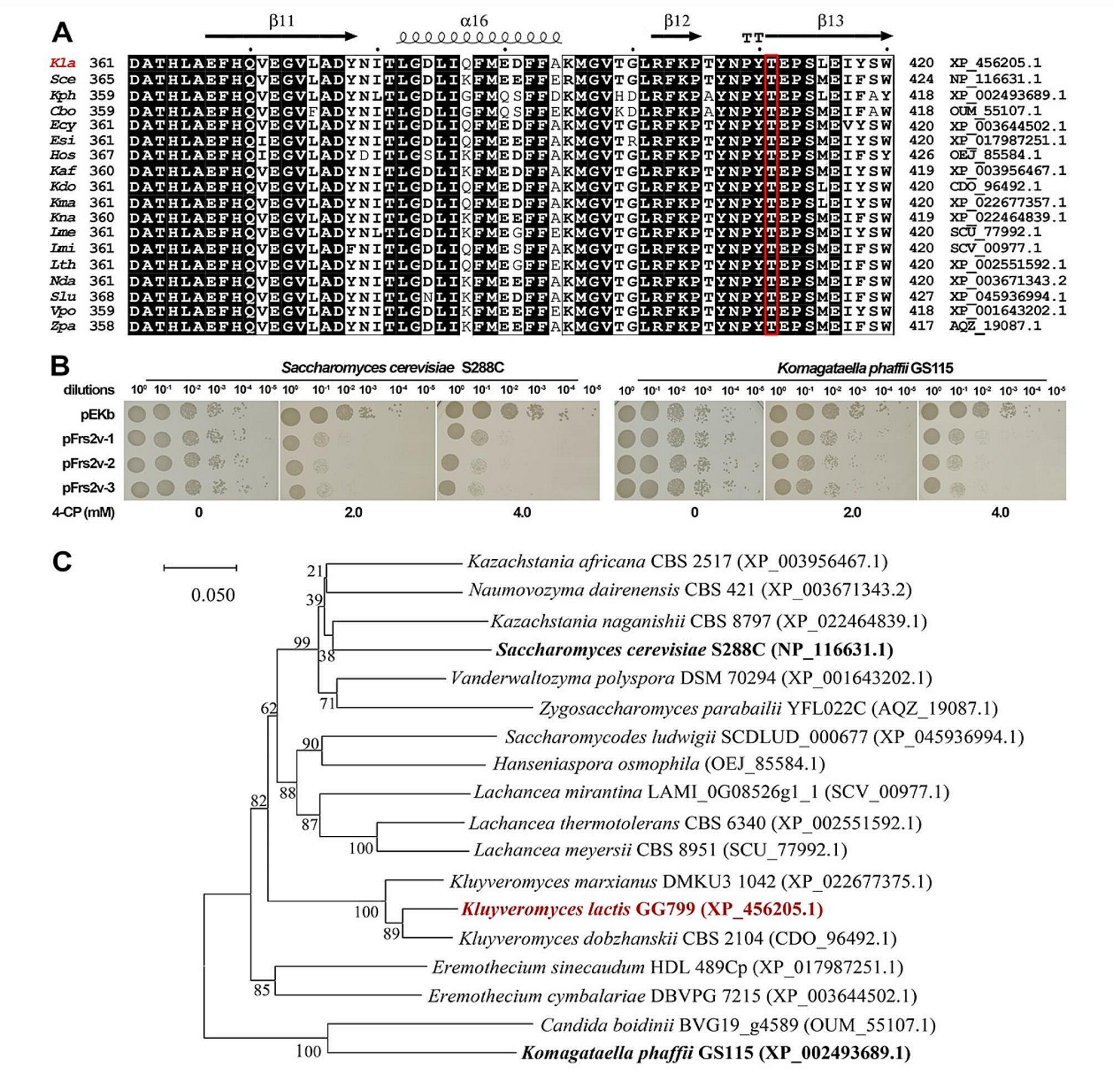
Application of the Frs2v from *K. lactis* for counterselection in *S. cerevisiae* and *K. phaffii*. **(A)** Multiple sequence alignment of Frs2 orthologs derived from yeasts, including *K. lactis* GG799 (*Kla* shown in red), S. cerevisiae S288C (*Sce*), *K. phaffii* GS115 (*Kph*), *Candida boidinii* BVG19_g4589 (*Cbo*), *Eremothecium cymbalariae* DBVPG 7215 (*Ecy*), *E. sinecaudum* HDL 489Cp (*Esi*), *Hanseniaspora osmophila* (*Hos*), *Kazachstania Africana* CBS 2517 (*Kaf*), *K. dobzhanskii* CBS 2104 (*Kdo*), *K. marxianus* DMKU3 1042 (*Kma*), *K. naganishii* CBS 8797 (*Kna*), *Lachancea meyersii* CBS 8951 (*Lme*), *L. mirantina* LAMI_0G08526g1-1 (*Lmi*), *L. thermotolerans* CBS 6340 (*Lth*), *Naumovozyma dairenensis* CBS 421 (*Nda*), *Saccharomycodes ludwigii* SCDLUD_000677 (*Slu*), *Vanderwaltozyma polyspora* DSM 70,294 (*Vpo*), and *Zygosaccharomyces parabailii* YFL022C (*Zpa*). The T411 residual of the *K. lactis* Frs2 and the corresponding T residuals in other Frs2 proteins are indicated with a red box. (**B**) 4-CP sensitivity of *S. cerevisiae* and *K. phaffii* cells with or without a Frs2v-expressing plasmid. Cell cultures of transformants of each strain either harboring the pEKb vector, or the Frs2v-expressing plasmid pFrs2v, were serially 10-fold diluted. Dilutions were spotted onto YPD plates containing 4-CP at the indicated concentrations. (**C**) Neighbor-joining phylogenetic tree based on amino acid sequences of Frs2 orthologs showing the phylogenetic relationship of the Frs2 from *K. lactis* GG799 (shown in red fonts) and that from the selected yeast species

Amino acid sequence alignment of Frs2 from *K. lactis* with several Frs2 homologues from other yeasts was performed, revealing their extremely high overall identity (Fig. 4A). Given this observation, the Frs2v-mediated counterselection might be generally applicable in many yeast species. For initial confirmation, we tested the counterselection capability of the Frs2v in two commonly used yeast strains, i.e. *S. cerevisiae* S288C and *K. phaffii* GS115. Results showed that cells of both yeasts expressing Frs2v were highly sensitive to 4-CP, although the lowest concentrations for growth inhibition of each yeast varied (Fig. 4B). Interestingly, according to the constructed phylogenetic tree of the Frs2 proteins, the enzymes from *K. lactis* GG799 and *K. phaffii* GS115 fell in distinct clades away apart (Fig. 4C). This was suggestive of that such Frs2v-based counterselection could be applicable in a broader range of yeast hosts.

## Conclusions

A counterselectable system based on a variant of the Frs2 protein derived from *K. lactis* GG799 was established, which not only worked in *K. lactis* to help attain efficient plasmid curing and scarless chromosomal engineering, but also was practicable in other yeasts such as the commonly applied *S. cerevisiae* and *K. phaffii*, providing a versatile tool for genetic manipulations of yeasts. Given the fact that no prior engineering of the host cells was required at all, it would represent one of currently the most convenient broad-host counterselection systems to allow for the speed-up of constructing yeast production platforms.

## Electronic supplementary material

Below is the link to the electronic supplementary material.

Supplementary Material 1

## Data Availability

The authors declare that the main data supporting the findings of this work are available within the article and its supplementary information files or from the corresponding authors upon reasonable request.

## Data availability

No datasets were generated or analysed during the current study.
