# Supplementary material for: A convenient broad-host counterselectable system endowing rapid genetic manipulations of Kluyveromyces lactis and other yeast species
Source: Microb Cell Fact. 2024 Jul 26;23:212. doi: 10.1186/s12934-024-02488-w (PMC11282862; doi:10.1186/s12934-024-02488-w)
Supplement: Supplementary file 1 — Supplementary Material 1 [file 12934_2024_2488_MOESM1_ESM.doc]

**Table S1**. Oligonucleotides used in this work. Restriction sites or protruding nucleotides underlined; mutations highlighted in bold; sequences overlapped with plasmid shown as italics.

| **Oligonucleotide** | **Sequence (**5’-3’**)** |
| --- | --- |
| Padh1-Frs2v-pEKb-F | CCCTTAATTAAACTAGTTCTAGAACTGTAGCCCTAGACTTGAT |
| pEKb-Padh1-Frs2v-R | AGAACTAGTTTAATTAAGGGAAATGTGTAGTGCTGATTATGA |
| Frs2v-Padh1-F | AATCAACTATCTCATATACAATGTCTGATATTCAATTGGA |
| Padh1-Frs2v-R | TCCAATTGAATATCAGACATTGTATATGAGATAGTTGATT |
| Frs2v-(T411A)-F | AACCCATAC**GCT**GAACCTTCATTGGAAATTTA |
| Frs2v(T411A)-R | TGAAGGTTC**AGC**GTATGGGTTATAGGTTGGTT |
| Frs2v-pEKb-F | CCTTAATTAAACTAGTTCTAGAACAGAACTAAATGATAAACT |
| pEKb-Frs2v-R | TCTAGAACTAGTTTAATTAAGGGAAATGTGTAGTGCTGATT |
| Frs2v-pEKb-R | AGGCTTTAATTTGCAAGCTTGAATTCTAGTCTGTATAGATTACTAT |
| pEKb-Frs2v-F | AGACTAGAATTCAAGCTTGCAAATTAAAGCCTTCGAGCGT |
| pEKb-gfp-F | *TTAATTAAACTAGTTCTAGA*CAGACCGAAGGAACTACTGG |
| gfp-pEKb-R | *TCTAGAACTAGTTTAATTAA*GGGAAATGTGTAGTGCTGAT |
| Frs2v-F | CCTGGTACCATAGCAGGATG*ACTGTAGCCCTAGACTTGAT* |
| gfp-R | *ATCAAGTCTAGGGCTACAGT* |
| G-ARM-F | ATGAGCTCTATTACCTCACCT |
| BLEOR-G-ARM-R | *CATTATCACCAAGCTCTTACTGC*TACAGAGAATGAACATGCTCC |
| G-ARM-BLEOR-F | GCGACGGTATCCACGTGCAGAAC |
| FRS2V-BLEOR-R | *GCTATCAAGTCTAGGGCTACAGT*ACGGGAAGTCTTTACAGTTT |
| BLEOR-FRS2V-F | ACTGTAGCCCTAGACTTGATAGCC |
| L-arm-Frs2v-R | *GAAAGGAGTGAATCAAACTG*AACATACAAAGAGACAGGC |
| Frs2v-L-arm-F | CAGACCGAAGGAACTACTGG |
| gfp-L-arm-R | *CGCCCTTGCTCACCAT*AGTTCAAAAAATATCCACC |
| L-arm-gfp-F | *ATGGTGAGCAAGGGCG*AGGAGCTGTTC |
| R-arm-gfp-R | *GATCTAAGTAACAATGAAC*TCACTTGTACAGCTCGTCCATGCC |
| gfp-R-arm-F | *GTTCATTGTTACTTAGATC*GTTTTATTTC |
| R-arm-R | CATCCTGCTATGGTACCAGGTAC |
| chk-fwd | TGTCTCAAATTACTCGACGGC |
| chk-rev | CCTTTCGAGGTCTTCCCAACAT |

**Table S2**. Complete sequences of the *E. coli*-*K. lactis* shuttle vector pEKb, and the pFrs2v plasmid.

| **Plasmid** | | **Sequence** | |
| --- | --- | --- | --- |
| pEKb | gggtctgacgctcagtggaacgaaaactcacgttaagggattttggtcatgagattatcaaaaaggatcttcacctagatccttttaaattaaaaatgaagttttaaatcaatctaaagtatatatgagtaaacttggtctgacagcaacatctttggataatatcagaatgagaaagaacagatacgcagtacgttttttggtgagctctttgcacttctttagttctttccatcaatatcagttgcttatgcacttatgactaatattgatgtttaacttcaatatctttaaacttttgttcttcccgacgttcattaagaatactaatacactttaataattagtttaatatttgtttctatataatgacatttaattaaaaaagataaaatataaaaacatcataataactcaccagaggttaagaacaaaaaaacaaattagatatctgctaatccaatatagttaaatcaatctttccttggtataatgggtatattacatatatttcaaggaccgacactcctaccaaatatctaaaatttaccatattaacataacatgtatataaacgtcaaatcataatcagcactacacatttcccttaattaaactagttctagactcgagaggcctccatggctgcaggaattcaagcttgcaaattaaagccttcgagcgtcccaaaaccttctcaagcaaggttttcagtataatgttacatgcgtacacgcgtctgtacagaaaaaaaagaaaaatttgaaatataaataacgttcttaatactaacataactataaaaaaataaatagggacctagacttcaggttgtctaactccttccttttcggttagagcggatgtggggggagggcgtgaatgtaagcgtgacataactaattacatgatatcgacaaaggaaaagggggacggatctccgaggcctgggacccgtgggccgccgtcggacgtgtcagtcctgctcctcggccacgaagtgcacgcagttgccggccgggtcgcgcagggcgaactcccgcccccacggctgctcgccgatctcggtcatggccggcccggaggcgtcccggaagttcgtggacacgacctccgaccactcggcgtacagctcgtccaggccgcgcacccacacccaggccagggtgttgtccggcaccacctggtcctggaccgcgctgatgaacagggtcacgtcgtcccggaccacaccggcgaagtcgtcctccacgaagtcccgggagaacccgagccggtcggtccagaactcgaccgctccggcgacgtcgcgcgcggtgagcaccggaacggcactggtcaacttggccatggtttagttcctcaccttgtcgtattatactatgccgatatactatgccgatgattaattgtcaacaccgcccttagattagattgctatgctttctttctaatgaacaagaagtaaaaaaagttgtaatagaacaagaaaaatgaaactgaaacttgagaaattgaagaccgtttattaacttaaatatcaatggaggtcactgaaagagaaaaaaactaaaaaaaaaaatttcaagaaaaagaaacgtgataaaaatttttattgcctttttcgacgaagaaaaagaaacgaggcggtctcttttttcttttccaaacctttagtacgggtaattaacgacaccctagaggaagaaagagggaaaatttagtatgctgtgcttgggtgttttgaagtggtacggcgatgcgcggagtccgagaaaatctggaagagtaaaaaaggagtagaaacattttgaagctatggtgtgtgggggatcggatcccatatgcagctgtcgacaattaatatttacttattttggtcaaccccaaataggttgatttcatacttggttcattcaaaaataagtagtcttttgagatctttcaatattataataaatatactataacagccgacttgtttcattttcgcgaatgttcccccagcttatctcgagcggccgcgaaagaacatgtgagcaaaaggccagcaaaaggccaggaaccgtaaaaaggccgcgttgctggcgtttttccataggctccgcccccctgacgagcatcacaaaaatcgacgctcaagtcagaggtggcgaaacccgacaggactataaagataccaggcgtttccccctggaagctccctcgtgcgctctcctgttccgaccctgccgcttaccggatacctgtccgcctttctcccttcgggaagcgtggcgctttctcatagctcacgctgtaggtatctcagttcggtgtaggtcgttcgctccaagctgggctgtgtgcacgaaccccccgttcagcccgaccgctgcgccttatccggtaactatcgtcttgagtccaacccggtaagacacgacttatcgccactggcagcagccactggtaacaggattagcagagcgaggtatgtaggcggtgctacagagttcttgaagtggtggcctaactacggctacactagaagaacagtatttggtatctgcgctctgctgaagccagttaccttcggaaaaagagttggtagctcttgatccggcaaacaaaccaccgctggtagcggtggtttttttgtttgcaagcagcagattacgcgcagaaaaaaaggatctcaagaagatcctttgatcttttctacg | |  |
| pFrs2v | gggtctgacgctcagtggaacgaaaactcacgttaagggattttggtcatgagattatcaaaaaggatcttcacctagatccttttaaattaaaaatgaagttttaaatcaatctaaagtatatatgagtaaacttggtctgacagcaacatctttggataatatcagaatgagaaagaacagatacgcagtacgttttttggtgagctctttgcacttctttagttctttccatcaatatcagttgcttatgcacttatgactaatattgatgtttaacttcaatatctttaaacttttgttcttcccgacgttcattaagaatactaatacactttaataattagtttaatatttgtttctatataatgacatttaattaaaaaagataaaatataaaaacatcataataactcaccagaggttaagaacaaaaaaacaaattagatatctgctaatccaatatagttaaatcaatctttccttggtataatgggtatattacatatatttcaaggaccgacactcctaccaaatatctaaaatttaccatattaacataacatgtatataaacgtcaaatcataatcagcactacacatttcccttaattaaactagttctagaactgtagccctagacttgatagccatcatcatatcgaagtttcactaccctttttccatttgccatctattgaagtaataataggcgcatgcaacttcttttctttttttttcttttctctctcccccgttgttgtctcaccatatccgcaatgacaaaaaaatgatggaagacactaaaggaaaaaattaacgacaaagacagcaccaacagatgtcgttgttccagagctgatgaggggtatctcgaagcacacgaaactttttccttccttcattcacgcacactactctctaatgagcaacggtatacggccttccttccagttacttgaatttgaaataaaaaaaagtttgctgtcttgctatcaagtataaatagacctgcaattattaatcttttgtttcctcgtcattgttctcgttccctttcttccttgtttctttttctgcacaatatttcaagctataccaagcatacaatcaactatctcatatacaatgtctgatattcaattggaaattttacagaaagtcgaaaagttgggtcaaattgactcaacctctgagctgtaccccagtatcgattctcaaaccatggctgcttatttgaattctttgaaatcccatgaaaagatcgatttctcaaagaaagataccattttctacagtttaactaaagaaggtaatgcgatcgtggccgatggttctcatgaagtgaaactcttgaaattgattgatcagtttggaaaattgcaaatcaaagatgttgccagtcatctaggcgccgacgggaaggttggtcaagctagagccttcaagaacggatggattgtaaagacaccagaaaatgaattgaaagttagtgacaaaattgcagatgtctcccaagtgaaagatcaaaccaaggaacaattggaaaagattaagaataacgaactttctggtatcagtgataaagaagttgctgatctaaagaagagaaagttaatcaccccaagaaaggaaactacattcagcgttgtcaagggcaaggaattctctaccgatttgactaagctggagactgagattaccgctgaaatggtagccacaggttcatataaggatctaaggttcaaagagttcaatttcaactctcaaggtatcgatccacaatccggtgctctgcatcctttgaacaaggtcagagaggaattcagacaaattttcttctctatgggtttcactgaaatgccatcaaaccaatacgtagaatcaggattctggaactttgacacattatacgttccacaacaacatccagctcgtgatttgcaagatacattttacttgaaggacccaatcaaatgcgatatgccagatgatagagaatatatcgccaacatcaaagctgtacacgagaatggtaaatttgattcgattggttaccgttacaactggaaggaggaagagtgccaaagattggttcttagaacacatactacagcaatatctgctgctatgcttcataagttggccaaagatccaaaaccaaccagattattctcgatagaccgtgtcttccgtaatgaagctgttgacgctacacatttagccgaattccaccaagttgaaggtgttttagctgattacaatataacgttgggtgacttaatccaattcatggaagacttcttcgctaagatgggtgttacggggttgcgtttcaaaccaacctataacccatacgctgaaccttcattggaaatttattcatggcatgaagggttaggcaaatgggttgagattggtaactccggtatgttcagaccagaaatgttggaatccatgggccttccaaaggatatgagagttctgggttggggtttgtctttagagagacctacaatgattaaatacaaggttcaaaatattagagagcttttgggtcacaaggtttctttggatttcattgaatcgaaccctgccgctaggttagatgaagatttgtatgaatgattttccttgattgttctaaattgcttttggtagtttataaaatctatatatgcgcttcatatcttcaaatcacctgtcagatagtaatctatacagactagaattcaagcttgcaaattaaagccttcgagcgtcccaaaaccttctcaagcaaggttttcagtataatgttacatgcgtacacgcgtctgtacagaaaaaaaagaaaaatttgaaatataaataacgttcttaatactaacataactataaaaaaataaatagggacctagacttcaggttgtctaactccttccttttcggttagagcggatgtggggggagggcgtgaatgtaagcgtgacataactaattacatgatatcgacaaaggaaaagggggacggatctccgaggcctgggacccgtgggccgccgtcggacgtgtcagtcctgctcctcggccacgaagtgcacgcagttgccggccgggtcgcgcagggcgaactcccgcccccacggctgctcgccgatctcggtcatggccggcccggaggcgtcccggaagttcgtggacacgacctccgaccactcggcgtacagctcgtccaggccgcgcacccacacccaggccagggtgttgtccggcaccacctggtcctggaccgcgctgatgaacagggtcacgtcgtcccggaccacaccggcgaagtcgtcctccacgaagtcccgggagaacccgagccggtcggtccagaactcgaccgctccggcgacgtcgcgcgcggtgagcaccggaacggcactggtcaacttggccatggtttagttcctcaccttgtcgtattatactatgccgatatactatgccgatgattaattgtcaacaccgcccttagattagattgctatgctttctttctaatgaacaagaagtaaaaaaagttgtaatagaacaagaaaaatgaaactgaaacttgagaaattgaagaccgtttattaacttaaatatcaatggaggtcactgaaagagaaaaaaactaaaaaaaaaaatttcaagaaaaagaaacgtgataaaaatttttattgcctttttcgacgaagaaaaagaaacgaggcggtctcttttttcttttccaaacctttagtacgggtaattaacgacaccctagaggaagaaagagggaaaatttagtatgctgtgcttgggtgttttgaagtggtacggcgatgcgcggagtccgagaaaatctggaagagtaaaaaaggagtagaaacattttgaagctatggtgtgtgggggatcggatcccatatgcagctgtcgacaattaatatttacttattttggtcaaccccaaataggttgatttcatacttggttcattcaaaaataagtagtcttttgagatctttcaatattataataaatatactataacagccgacttgtttcattttcgcgaatgttcccccagcttatctcgagcggccgcgaaagaacatgtgagcaaaaggccagcaaaaggccaggaaccgtaaaaaggccgcgttgctggcgtttttccataggctccgcccccctgacgagcatcacaaaaatcgacgctcaagtcagaggtggcgaaacccgacaggactataaagataccaggcgtttccccctggaagctccctcgtgcgctctcctgttccgaccctgccgcttaccggatacctgtccgcctttctcccttcgggaagcgtggcgctttctcatagctcacgctgtaggtatctcagttcggtgtaggtcgttcgctccaagctgggctgtgtgcacgaaccccccgttcagcccgaccgctgcgccttatccggtaactatcgtcttgagtccaacccggtaagacacgacttatcgccactggcagcagccactggtaacaggattagcagagcgaggtatgtaggcggtgctacagagttcttgaagtggtggcctaactacggctacactagaagaacagtatttggtatctgcgctctgctgaagccagttaccttcggaaaaagagttggtagctcttgatccggcaaacaaaccaccgctggtagcggtggtttttttgtttgcaagcagcagattacgcgcagaaaaaaaggatctcaagaagatcctttgatcttttctacg | |  |
